# Analytical insight into degradation processes of aminopolyphosphonates as potential factors that induce cyanobacterial blooms

**DOI:** 10.1007/s11356-017-0068-1

**Published:** 2017-09-10

**Authors:** Damian Drzyzga, Jacek Lipok

**Affiliations:** Faculty of Chemistry, Opole University, Oleska 48, 45-052 Opole, Poland

**Keywords:** Aminopolyphosphonates, Pollutant transformation, Analytical determination, Cyanobacterial biodegradation, Water pollution, HPLC

## Abstract

Aminopolyphosphonates (AAPs) are commonly used industrial complexones of metal ions, which upon the action of biotic and abiotic factors undergo a breakdown and release their substructures. Despite the low toxicity of AAPs towards vertebrates, products of their transformations, especially those that contain phosphorus and nitrogen, can affect algal communities. To verify whether such chemical entities are present in water ecosystems, much effort has been made in developing fast, inexpensive, and reliable methods for analyzing phosphonates. However, unfortunately, the methods described thus far require time-consuming sample pretreatment and offer relatively high values of the limit of detection (LOD). The aim of this study was to develop an analytical approach to study the environmental fate of AAPs. Four phosphonic acids, *N,N*-bis(phosphonomethyl)glycine (GBMP), aminotris(methylenephosphonic) acid (ATMP), hexamethylenediamine-*N,N,N′,N′-*tetrakis(methylphosphonic) acid (HDTMP), and diethylenetriamine penta(methylenephosphonic) acid (DTPMP) were selected and examined in a water matrix. In addition, the susceptibility of these compounds to biotransformations was tested in colonies of five freshwater cyanobacteria—microorganisms responsible for the so-called blooms in the water. Our efforts to track the AAP decomposition were based on derivatization of *N*-alkyl moieties with *p*-toluenesulfonyl chloride (tosylation) followed by chromatographic (HPLC-UV) separation of derivatives. This approach allowed us to determine seven products of the breakdown of popular phosphonate chelators, in nanomolar concentrations and in one step. It should be noted that the LOD of four of those products, aminemethylphosphonic acid (AMPA), *N-*phosphomethyl glycine (NPMG), *N*-(methyl)aminemethanephosphonic acid (MAMPA), and *N-*(methyl) glycine (SAR), was set below the concentration of 50 nM. Among those substances, *N*-(methylamino)methanephosphonic acid (MAMPA) was identified for the first time as the product of decomposition of the examined aminopolyphosphonates.

## Introduction

Demand for organophosphonates has intensified in recent years because thousands of tons of these compounds are consumed by industry, agriculture, and households each year (Benbrook 2016, Coupe et al. 2012, Studnik et al. 2015). Consequently, the environmental consequence of this is the annual introduction of several thousand tons of aminopolyphosphonates (AAPs), which are released mainly into surface waters (Studnik et al. 2015). Among all phosphonates, glyphosate (*N-*phosphonomethyl glycine) is applied in millions of kilograms per year worldwide, and its main metabolite AMPA (aminomethylphosphonic acid) appears as the most important contaminant and is accompanied by identification of toxic effects of glyphosate on some water organisms (Pesce et al. 2009, Vendrell et al. 2009). For example, *N-*phosphonomethyl glycine and AMPA have been detected in Mississippi River basin at levels above 0.1 μg L^−1^ (Coupe et al. 2012). Additionally, glyphosate accumulation in tissues of some water organisms, including *Daphnia pulex*, snails (*Helix aspersa*), carp (*Cyprinus carpio*), and tilapia (*Oreochromis mossambicus*) (Annett et al. 2014), have been reported. In 2015, glyphosate was classified as a “probable human carcinogen” by the International Agency for Research on Cancer (IARC 2015).

In comparison with glyphosate and AMPA, the knowledge on environmental fate and ecological interactions of aminopolyphosphonates that are commonly used as complexones of metal ions and as flame retardants is very scarce. More surprising is that the worldwide demand for these compounds reaches millions of tons annually (Studnik et al. 2015). Aminopolyphosphonates possess a nitrogen atom to which methylphosphonic motifs are anchored. Therefore, it is expected that such complexones may be, in a way, similar to glyphosate. Microbial biodegradation of such substances was reported with respect to mycelial fungus *Aspergillus terreus* (Lenartowicz et al. 2015) and cyanobacteria (Forlani et al. 2013, 2008, 2011, Lipok et al. 2010), with aminopolyphosphonates serving as the source of nutritive phosphorus for these microorganisms. Obviously, the patterns of decomposition of individual compounds vary depending on their structure (Drzyzga et al. 2017). Some of these compounds have been reported as being sensitive to chemical decomposition in water, in the presence of Fe(III) and Mn(II) ions, which serve as catalysts (Nowack 2003). However, their decomposition pathways have not been studied.

Independent of the degradation method, phosphorus that is present in structures of phosphonates enriches total P content in water reservoirs, which leads to their eutrophication (Sharpley and Wang 2014). The U.S. Environmental Protection Agency described this phenomenon as one of the main problems that impair water quality (US Environmental Protection Agency 2011). Frequently, this results in blooms of cyanobacteria and harmful algae. Cyanobacteria, also called blue-green algae, are the most widely distributed groups of photosynthetic prokaryotes on the Earth and are the pioneering organisms that are present in almost all defined ecosystems of the planet, mostly inhabiting surface waters (Schopf 2000). Cyanobacteria, as key organisms in environmental nutrition cycling (Benitez-Nelson 2015, Heimann and Cires 2015), may respond to the presence of phosphonates by decreasing their environmental abundance through their conversion into phosphates. The precise environmental fate of these xenobiotics, however, is unknown. Currently, phosphonates (including aminopolyphosphonates) that participate in the global phosphorus cycle can be incepted by microorganisms (Van Mooy et al. 2015). Furthermore, it is important to define the routes of transformation of aminopolyphosphonates and the fate of these compounds. However, such studies are hampered by the lack of efficient analytical methods that allow tracking the chemical changes of substrate molecules.

Many techniques and procedures have been developed and successfully used, mainly in the analysis of glyphosate and AMPA: gas chromatography (GC/MS, GC/MS/MS), high-performance liquid chromatography (HPLC), capillary electrophoresis (CE), and enzyme-linked immunosorbent assay (ELISA) (Annett et al. 2014, Poiger et al. 2017, Stalikas and Konidari 2001, Toss et al. 2017). Recently, the attempts to monitor glyphosate presence using various sensors, such as supramolecular, molecular imprinted polymers, nanofibre ones, quantum dots, or conjugated polymer-based fluorescent (CPF) chemosensors, have shown great potential in detecting this compound (Gui et al. 2017). Although the analysis of other phosphoric pesticides or phosphonic derivatives has been reported (Skeff et al. 2016, Stalikas et al. 2000), despite the use of various detection and derivatization techniques (Koskinen et al. 2016), the analysis of aminophosphonic acids in water is still extremely difficult. The main reasons are their hydrophilic character and the lack of specific chromophore in their structures (Nowack 2003). High water solubility with simultaneous poor solubility in organic solvents makes phosphonic acid derivatives extremely hard to extract from environmental matrices (Stalikas and Konidari 2001). Many efforts have been undertaken to develop fast, inexpensive, and reliable methods for their analysis (Esser et al. 2007, Nowack 1997, 2002, 2003) but without spectacular success. The LOD values of those methods were above 1 μM, which makes them unsuitable for application to natural samples. The application of phosphorus nuclear magnetic resonance (^31^P NMR) allowed tracing the fate of glyphosate in a culture of cyanobacterium *Spirulina platensis* (Lipok et al. 2007). However, the use of this technique in complex matrices is confined by high detection limits, which are additionally increased in the presence of paramagnetic metal ions in the samples.

Bearing in mind the abovementioned information, it can be stated that increasing and uncontrolled loading of aminophosphonates into the environment, combined with the ability of cyanobacteria to transform these compounds, resulted in the appearance of AAP degradation products in the environment. Unfortunately, there is a lack of a convenient method for simultaneous determination of such derivatives. Therefore, we focused on three aspects when studying the fate of aminopolyphosphonates: (i) to adapt and to improve the already existing HPLC-UV procedure for the determination of glyphosate and AMPA in water (Khrolenko and Wieczorek 2005) to make it useful to study the presence of a wider spectrum of aminophosphonates, (ii) to track stability of these compounds in water and in media upon culturing several cyanobacteria strains, and (iii) to follow the processes of aminopolyphosphonate (bio)degradation. In this respect, interactions between four aminopolyphosphonates with five freshwater cyanobacteria species were assessed, using a relatively simple, inexpensive, and fast procedure, which allows detection of these biodegradation products even at a nanomolar level.

## Experimental

### Reagents

All chemicals, if not specified, were purchased from Avantor Performance Materials Poland S.A. (Gliwice, Poland). Water that was used to prepare all solutions was purified using the Milli-Q-RO4 system (Millipore, Bedford, MA, USA). In addition to the components of cyanobacterial media, which were of technical grade, all chemicals were of analytical grade.

#### Aminopolyphosphonates

Aminopolyphosphonates (Table 1) were obtained from Zschimmer & Schwarz GmbH & Co KG (Mohsdorf, Germany), a leading European producer of phosphonates.

**Table 1:**
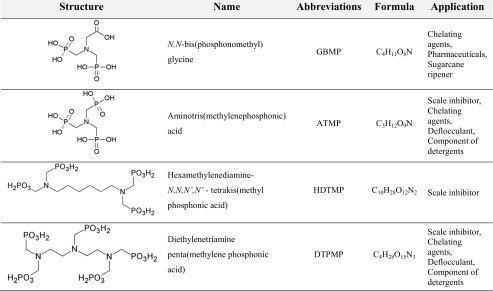
Studied aminopolyphosphonates with a list of their practical applications based on Studnik (Studnik et al. 2015) and manufacturer’s data

#### Potential products of decomposition of aminopolyphosphonates

Considering that the tested aminopolyphosphonates contain a common motif (−N-CH_2_-P(O)(OH)_2_), it may be expected that degradation processes will result in the formation of common intermediates and/or final products. In Table 2, seven of such intermediates that contain amine functions were proposed as common intermediates in the processes of (bio)degradation of compounds that are presented in Table 1. They were selected based on the pathways of microbial degradation of glyphosate (Lipok et al. 2007, Sviridov et al. 2015).

**Table 2:**
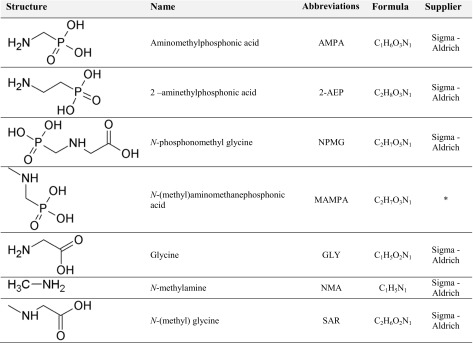
Postulated products and intermediates of aminopolyphosphonate decomposition. Asterisk indicates this compound was a kind gift from Dr. Marta Bochno PhD, and its synthesis is described in (Berlicki et al. 2012)

Standard stock solutions (1 mM) of presumable products of degradation of aminopolyphosphonates were prepared in a sterile Bg11 medium and were stored in the dark at 4 °C. Analytical solutions were prepared by diluting stock solutions with a sterile Bg11 medium just before the analysis.

### Determination of stability of aminopolyphosphonates

The solutions of tested compounds at concentrations of 0.1 mM were prepared in sterile (autoclaved) Milli-Q water as well as in a Bg11 (cyanobacterial) medium. To maintain sterile conditions, appropriate amounts of each aminopolyphosphonate were dissolved in a few milliliters of sterile medium and then added to the final test solutions via filtration through a sterile membrane syringe filter (Nylon, 0.22 μm). Thus, the obtained sterile solutions of aminopolyphosphonates in media (50 mL) were placed in sterile 250-mL Erlenmeyer flasks and maintained for at least 14 days at the same conditions as all other experiments, therefore acting as substrate controls.

### Sample collection and derivatization

The 1 mL samples of experimental cultures of cyanobacteria, as well as the samples of substrate controls, were collected at 3–4-day intervals and were frozen in liquid nitrogen and stored at − 28 °C. On the day of analysis, appropriate samples were thawed and subjected to derivatization.

The derivatization procedure relied on tosylation of primary and secondary amine groups and was adapted from the procedure described by Khrolenko and Wieczorek (Khrolenko and Wieczorek 2005). Briefly, 150 μL of the sample solution was mixed with 75 μL of 0.4 M K_2_HPO_4_ (pH 11), and 30 μL of *p*-toluenesulfonyl chloride (10 mg mL^−1^ in MeCN) was added. Then, the reaction mixture was heated in water bath at 50 °C for 10 min. Derivatization was terminated by adding 32.6 μL of 1 M HCl to each sample.

### Chromatographic (HPLC) separation of analytes

The samples were analyzed using a Thermo Scientific Dionex Ultimate ® 3000 HPLC system, equipped with a Photodiode Array Detector (PDA-3000) that monitors the eluate at 240 nm. The derivatized samples (20 μL) were injected onto a 4.6 mm × 250 mm Phenomenex Kinetex ® Evo 100-5 C-18 column connected with a dedicated SecurityGuard ™ ULTRA Cartridge System. HPLC separations were performed using a mobile phase that consists of a 10 mM KH_2_PO_4_ buffer (adjusted to pH 2.3 with 85% H_3_PO_4_) and acetonitrile with the initial ratio of 90:10 (*v*/*v*), respectively. During the first 17 min, the composition of the mobile phase was unchanged, but the flow rate increased from 1.0 mL min^−1^ and reached the maximum of 1.3 mL min^−1^ at 17 min. From 17 to 24 min, the contribution of phosphate buffer was reduced to 75% (2.14% min^−1^), which was accompanied by the decrease in the flow rate to 1.0 mL min^−1^. Next, during the time from 24 to 31 min of analysis, the composition of the mobile phase was gradually returned to initial parameters with a similar velocity, with the flow rate of 1.0 mL min^−1^. These conditions were maintained up to the 36th minute, when the separation was finished. Elution proceeded at 35 °C. The samples of stock solutions were run with a six-fold repetition, whereas experimental samples were analyzed at least in triplicate.

### Validation of the analytical procedure

The following features of the HPLC-PDA method, such as linearity, limit of detection (LOD), limit of quantitation (LOQ), and precision, were investigated and described. Validation was performed both in a fresh Bg11 medium and in a post-cultured BG11 medium. LOD and LOQ were computed based on the standard deviation of the response and slope (LOD = 3.3 *σ*/*s*, where *σ*—S.D. of response, *s*—slope of calibration curve). Limit of quantification (LOQ) was expressed as a three-fold of LOD or as LOQ = 10 *σ*/*s*. Additionally, the impact of freezing stock solutions on LOD and LOQ values was assessed. On the day of analysis, the stock solution mixtures of *N*-containing compounds were prepared in a fresh, sterile Bg11 medium at a concentration of 1 mM. The same Bg11 medium was used to prepare a set of solutions of the tested compounds and products at a desired concentration range. All prepared solutions were frozen in liquid nitrogen and maintained at − 28 °C for 7 days. Then, they were thawed and analyzed again. Post-cultured medium was applied as a matrix. It was prepared as follows. After 14 days of culturing freshwater cyanobacterium *Anabaena variabilis* in a Bg11 medium, microbial cells were removed via centrifugation (5000×*g*/5 min), and the standards were dissolved in the obtained supernatant to reach the final concentration of 1 mM. Post-cultured Bg11 medium was applied to dilute the stock solutions, similar to the fresh Bg11 medium. The calibration curves were constructed based on six to eight points, with six injections for each concentration of each standard solution.

### Cyanobacterial cultures

Cyanobacterial strains were obtained from the Culture Collection of Autotrophic Organisms at the Institute of Botany of the Academy of Sciences of the Czech Republic. The following species were examined: *Anabaena variabilis* (CCALA 007), *Chroococcidiopsis thermalis* (CCALA 049), *Chroococcus minutus* (CCALA 055), *Fischerella* cf. *maior* (CCALA 067), and *Nostoc* cf. *muscorum* (CCALA 129). These strains have been chosen due to their participation in cyanobacteria blooms of water bodies. Microorganisms were grown at 25 ± 1 °C under 16 h:8 h (day:night) in 250-mL Erlenmeyer flasks containing 60 mL of a Bg11 medium (ATCC 616) (Allen 1968, Rippka 1979). To maintain cell vitality, every 21 days, the cultures were revitalized (by transferring 10-mL aliquots to 50 mL of fresh medium). Experimental cultures were started in 50 mL of solutions of aminopolyphosphonate in a Bg11 medium, followed by the addition of cells of cyanobacteria to obtain the final concentrations of chlorophyll of 1 mg L^−1^. The chlorophyll content was determined using the already described method (Lipok et al. 2010).

To assess whether the presence of inorganic phosphate can affect the explanation of the AAP stability and the microbial ability to degrade inorganic phosphate, additional experiments were performed. A standard Bg11 medium was modified by removing K_2_HPO_4_ (Bg11-P); in this case, it was necessary to balance the potassium concentration by providing it in the form of KNO_3_ (34 mg L^−1^), which resulted in the reduction of the amount of NaNO_3_ to 1.47 g L^−1^ (to balance the amount of N).

## Results and discussion

### Adaptation and optimization of the HPLC-based determination procedure of amino derivatives

**Fig. 1:**
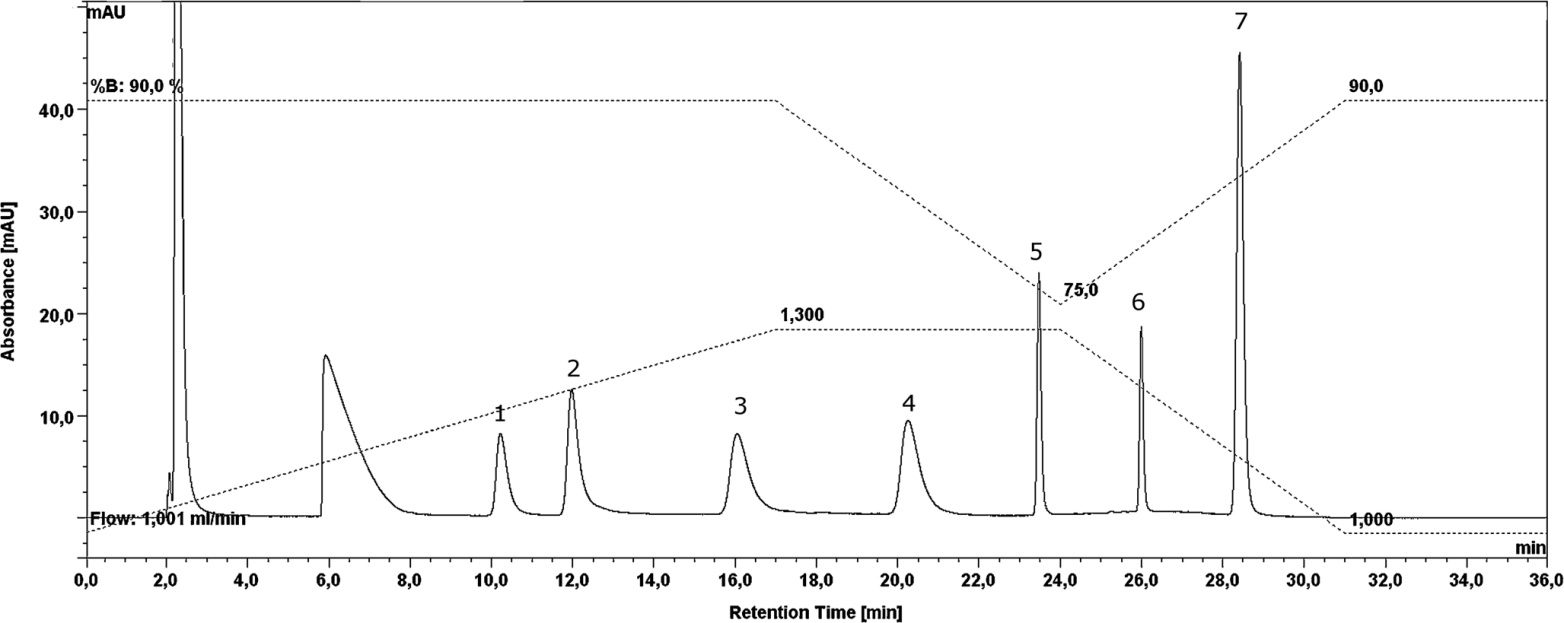
Chromatogram, which presents the final separation of all seven compounds postulated as (bio)degradation products applied at a concentration of 100 μM and introduced to the 14-day-old post-cultured Bg11 medium. Amine compounds, in the form of tosyl derivatives, were separated in the following order: 1—AMPA; 2—2-AEP; 3—NPMG; 4—MAMPA; 5—Gly; 6—NMA; 7—SAR. The chromatogram also contains the conditions of separation marked as a dotted line. The lower one corresponds to the flow rate, while the upper one presents time-dependent participation of a KH_2_PO_4_ buffer in a mobile phase composition, with respect to MeCN. The unnumbered peak with a retention time of approximately 6 min is attributed to *p*-toluenesulfonyl chloride

Because the application of HPLC chromatographic conditions, which have been already described, did not allow effective separation of all seven predicted intermediates or final products of AAP degradation; several factors, such as the flow rate, mobile phase composition, and column temperature, were optimized. The introduction of two gradient domains to isocratic separation [the first one after 17 min, which coincides with the stable flow of mobile phase (1.3 mL min^−1^), and the second from 24 to 31 min, accompanied by a gradual decrease of the flow (from 1.3 to 1.0 mL min^−1^)] allowed the reduction of the entire time of HPLC separation to approximately 36 min. The temperature of the column was set at 35 °C and was stable during the entire process. As shown in Fig. 1, the optimized conditions assured the successful, simultaneous separation of all seven presumable products of degradation in one step.

### Validation of the analytical procedure

- Linearity

Calibration curves were constructed based on analytical values obtained for aminopolyphosphonates dissolved in a fresh, sterile Bg11 medium, after 1 week of freezing of the samples, and in fresh and frozen 14-days post-cultured media. That approach allowed us to investigate both the impact of freezing and storage of samples at − 28 °C and the possible interference of analytes with microbial metabolites that are present in a post-culture media. Calibration curves for aminomethylphosphonic acid (AMPA), *N*-(methyl)aminomethanephosphonic acid (MAMPA), and *N-*phosphomethyl glycine (NPMG) were found to be linear in the concentration range from 0.8 to 100 μM. For 2–aminethylphosphonic acid (2-AEP) and glycine (Gly), the curves were linear in the range of 1.5 up to 100 μM, while for *N*-methylamine (NMA) and *N-*(methyl) glycine (SAR), the linearity was observed from 0.4 to 100 μM. The values of limits of detection (LOD), limits of quantification (LOQ), and correlation coefficients are given in Table 3.

**Table 3:** Computed LOD, LOQ, and *R* ^2^ values of compounds considered potential products of aminopolyphosphonate (bio)degradation. LOD and LOQ are expressed as (μM)

Compound	Medium	LOD	LOQ	*R* ^2^
AMPA	Bg11	0.024	0.073	0.9999
	Bg11 (frozen)	0.026	0.077	0.9997
	Post-cultured Bg11	0.028	0.083	0.9996
2-AEP	Bg11	0.158	0.474	0.9998
	Bg11 (frozen)	0.149	0.447	0.9999
	Post-cultured Bg11	0.160	0.481	0.9998
NPMG	Bg11	0.044	0.132	1.0000
	Bg11 (frozen)	0.050	0.151	0.9997
	Post-cultured Bg11	0.049	0.147	1.0000
MAMPA	Bg11	0.035	0.104	0.9998
	Bg11 (frozen)	0.038	0.114	0.9999
	Post-cultured Bg11	0.045	0.135	0.9994
Gly	Bg11	0.112	0.337	0.9999
	Bg11 (frozen)	0.159	0.476	0.9994
	Post-cultured Bg11	0.132	0.395	0.9996
NMA	Bg11	0.111	0.332	0.9999
	Bg11 (frozen)	0.128	0.385	0.9997
	Post-cultured Bg11	0.119	0.357	0.9993
SAR	Bg11	0.041	0.121	0.9999
	Bg11 (frozen)	0.045	0.149	0.9999
	Post-cultured Bg11	0.051	0.153	0.9995

The table includes the values of limit of detection and limit of quantification [expressed in (μM)] that are computed for stock solutions prepared in a fresh Bg11 medium (Bg11), after 7 days of freezing (Bg11 frozen), and stock solutions made in a 14-day-old post-cultured medium of *Anabaena variabilis* with cells being removed prior to the measurement (post-cultured Bg11).

The impact of freezing or the presence of cyanobacterial metabolites on the limit of detection was slight. The presence of cyanobacterial matrix had evoked the changes in the level of determination of MAMPA and SAR (compared with a Bg11 stock solution). Nevertheless, this difference amounted in only circa 10 nM. Freezing, in turn, influenced the LOD values mostly in the case of Gly (almost 50 nM). Nevertheless, the values of LOD and LOQ that are presented in Table 3 are of the same order of magnitude and are in a nanomolar concentration range. It should be noted that AMPA, NPMG, MAMPA, and SAR can be detected below the concentration of 50 nM. In regard to 2-AEP, Gly, and NMA, the detection levels were estimated in the range of 120–130 nM. Considering the simplicity of the procedure used and the complexity of the matrix, in which the compounds have been detected, the described analytical procedure seems to considerably simplify their tracking in natural waters.

b) Precision of the implemented HPLC method

The precision of the HPLC method was expressed as the percent relative standard deviation (%RSD) and determined for 0.025-mM stock solutions of the mixture of examined compounds (Table 4). For all batches, the values were below 5%, which proved that neither the freezing process nor the presence of cyanobacterial metabolites substantially affected the precision of the HPLC-based procedure used.

**Table 4:** Representative precision for 0.025-mM stock solutions

Compound	%RSD Bg11 medium day 0	%RSD Bg11 medium (frozen)—7th day	%RSD post-culture medium *Anabaena variabilis*
AMPA	3.48	1.45	2.35
2-AEP	1.44	1.69	3.79
NPMG	2.15	4.55	3.66
MAMPA	3.89	2.35	1.07
Gly	1.93	4.15	2.44
NMA	3.36	2.32	2.57
SAR	3.00	1.66	2.46

Precision was expressed as the percent relative standard deviation for a statistically significant number of samples (*n* = 6)

### Stability of the tested aminopolyphosphonates in the examined liquids

The absence of direct methods of aminopolyphosphonate determination, particularly at the concentration level below 0.1 μM, compels us to express their stability as a formation of new compounds. Indisputably, the components of a Bg11 medium influenced the stability of tested aminophosphonates, which resulted in the formation of some of the postulated breakdown products (Table 5.). All examined phosphonates underwent chemical decomposition that is accompanied by the release of an aminomethylphosphonic acid (AMPA) molecule but with a varied intensity. The emergence of *N*-(methyl)aminomethanephosphonic acid (MAMPA) among other decomposition products of aminotris(methylenephosphonic) acid (ATMP) and *N,N*-bis(phosphonomethyl) glycine (GBMP) should be underlined, especially because this compound has not yet been deliberated as a product of the aminopolyphosphonate transformation processes. The vulnerability of aminopolyphosphonates to degradation in Bg11 medium was characterized by the number and concentration of the formed products. Based on these data, the following order of increasing stability of tested compounds, which correlates with a growing number of methylphosphonic groups in molecules, can be concluded as follows: GBMP < ATMP < HDTMP < DTPMP.

**Table 5:** The presence of degradation products of aminopolyphosphonates in water solutions (H_2_O) and in Bg11 medium (Bg11) after 2 weeks of storage at room temperature, expressed in micromoles per liter (μM)

Substrates	Postulated products							
	AMPA	2-AEP	NPMG	MAMPA	Gly	NMA	SAR	
ATMP	H_2_O	–	–	–	–	–	–	–
	Bg11	34.0 ± 1.0	–	–	2.9 ± 0.4	–	–	–
GBMP	H_2_O	27.5 ± 0.7	–	–	–	–	–	–
	Bg11	3.0 ± 0.3	–	–	44.5 ± 1.9	*–*	–	–
HDTMP	H_2_O	–	–	–	–	–	–	–
	Bg11	12.6 ± 1.2	–	–^a^	–	–	–	–
DTPMP	H_2_O	–	–	–	–	–	–	–
	Bg11	7.8 ± 2.2	–	–	–	–	–	–

–Not detected

^a^In the solution of HDTMP acid in a Bg11 medium, compound characterized by a retention time that is very close to NPMG, which was detected in relatively large amounts. This issue is discussed in the body of this text

In addition to GBMP, which is not stable in water, instability of aminopolyphosphonates may result from the presence of some transition metal ions, as has been already stated by Nowack. It has been proven that in the presence of Mn(II), some AAPs undergo oxidation, whereas the presence of ions of Fe(II) and Fe(III) leads to the formation of phosphonate complexes, which undergo photodegradation (Nowack 2003). The molecule of aminotri(methylenephosphonic) acid (ATMP) subjected to oxidation in the presence of manganese ions forms iminedimethylenephosphonic acid (IDMP) and *N*-formyl-iminodimethylenephosphonic acid (FIDMP) (Nowack 2002).

In contrast to multifaceted and time-consuming HPLC determination, in which 9-fluorenylmethyl chloroformate (FMOC) was applied as a derivatization agent (Nowack 2002), the method applied in this work did not allow us to determine the presence of iminodi(methylenephosphonic) acid (IDMP). Although, Mn(II) ions are present in a Bg11 medium at a concentration approximately 10 times lower than the tested phosphonates, and ATMP breakdown in the presence of manganese cations was observed for equimolar amounts of these substances (Nowack and Stone 2000), the formation of iminodiphosphonic derivatives cannot be disputed. In addition, Nowack and Stone (Nowack and Stone 2000) postulated that Zn(II) and Ca(II) ions considerably inhibit the reaction by competing for the phosphonate substrate. In our study, these ions are present in Bg11 medium at a final concentration of 0.765 μM and 0.24 mM, respectively, and the process of degradation still occurs. Although the pH of the Bg11 medium (~ 7.1) favors degradation via metal ion-catalyzed pathways, the same process can be hampered by other components of the medium such as EDTA and Cu(II) ions, which completely inhibit AAP decomposition via IDMP and FIDMP release. The appearance of an aminomethylphosphonic acid (AMPA) molecule in the solution of ATMP in a Bg11 medium (Table 5) can be related to the IDMP occurrence due to the chemical structure of the substrate. However, the composition of a Bg11 medium suppressed the appearance of AMPA with respect to GBMP. In this case, the final concentration of aminomethylenephosphonic acid was approximately nine-fold lower compared with its solution in water.

### Tendency of aminopolyphosphonates for biodegradation

Aminopolyphosphonates that are placed in a sole Bg11 medium have shown the tendency for spontaneous degradation, probably as a consequence of the action of transition metal ions. However, to understand the environmental fate of AAPs, it is necessary to assess their interactions with organisms that are responsible for the cycle of elements in nature. Certainly, cyanobacteria rank highly in this respect (Cottingham et al. 2015). The usefulness of several phosphonates as sources of phosphorus for aquatic microorganisms has been already proven (Drzyzga et al. 2017, Forlani et al. 2011, Ravi and Balakumar 1998, Studnik et al. 2015). Concerning the impact of cyanobacteria on the stability of AAPs, it is worth noting that among the detected intermediates, there are compounds that have been identified after 2 weeks of incubation in a sole Bg11 medium. Nevertheless, the concentrations of these substances in cyanobacterial cultures are significantly higher, even up to four- or five-fold in some cases (Table 6).

**Table 6:** Concentrations of (bio)degradation products of aminopolyphosphonates after 2 weeks of culturing with cyanobacteria

Substrates	Cyanobacteria strain	Medium	Concentration of postulated products (μM)						
			AMPA	2-AEP	NPMG	MAMPA	Gly	NMA	SAR
ATMP	*Anabaena* 007	Bg11	115.0 ± 1.7	–	–	3.2 ± 0.5	–	–	–
		Bg11-P	102.3 ± 8.5	–	–	2.4 ± 0.4	–	–	–
	*Chroococcidiopsis* 049	Bg11	41.3 ± 0.9	–	–	2.8 ± 0.2	–	–	–
		Bg11-P	126.8 ± 2.5	–	–	2.5 ± 0.3	–	–	–
	*Chroococcus* 055	Bg11	85.8 ± 7.6	–	–	2.9 ± 0.4	–	–	–
		Bg11-P	90.8 ± 2.8	–	–	3.6 ± 0.2	–	–	–
	*Fischerella* 067	Bg11	133.4 ± 12.4	–	–	3.4 ± 0.6	–	–	–
		Bg11-P	111.4 ± 4.3	–	–	3.9 ± 1.5	–	–	–
	*Nostoc* 129	Bg11	72.0 ± 5.6	–	–	3.8 ± 1.0	–	–	–
		Bg11-P	109.7 ± 6.1	–	–	3.3 ± 1.4	–	–	–
GBMP	*Anabaena* 007	Bg11	4.7 ± 0.5	–	1.8 ± 0.3	111.5 ± 9.2	–	–	–
		Bg11-P	5.4 ± 0.3	–	1.2 ± 0.1	67.3 ± 1.8	–	–	–
	*Chroococcidiopsis* 049	Bg11	8.3 ± 1.1	–	2.3 ± 0.3	155.8 ± 9.8	–	–	–
		Bg11-P	3.7 ± 1.0	–	1.0 0.3	63.3 ± 8.5	–	–	–
	*Chroococcus* 055	Bg11	5.2 ± 0.3	–	1.5 ± 0.3	82.8 ± 6.8	–	–	–
		Bg11-P	3.0 ± 1.0	–	1.0 ± 0.1	51.5 ± 1.2	–	–	–
	*Fischerella* 067	Bg11	6.2 ± 0.6	–	2.3 ± 0.2	108.6 ± 1.2	–	–	–
		Bg11-P	4.2 ± 0.1	–	1.2 ± 0.1	65.7 ± 0.7	–	–	–
	*Nostoc* 129	Bg11	9.5 ± 0.4	–	2.0 ± 0.2	114.5 ± 2.3	1.5 ± 0.1	1.1 ± 0.1	–
		Bg11-P	5.8 ± 0.2	–	1.2 ± 0.1	73.2 ± 3.4	–	–	–
HDTMP	*Anabaena* 007	Bg11	^a^	–	–	–	–	–	–
		Bg11-P	–	–	–	–	–	–	–
	*Chroococcidiopsis* 049	Bg11	1.8 ± 0.2	–	–	0.7 ± 0.1	–	–	–
		Bg11-P	–	–	–	–	–	–	–
	*Chroococcus* 055	Bg11	2.1 ± 0.3	–	–	0.5 ± 0.1	–	–	–
		Bg11-P	–	–	–	–	–	–	–
	*Fischerella* 067	Bg11	^a^	–	–	–	–	–	–
		Bg11-P	–	–	–	–	–	–	–
	*Nostoc* 129	Bg11	2.5 ± 0.1	–	–	–	–	–	–
		Bg11-P	–	–	–	–	–	–	–
DTPMP	*Anabaena* 007	Bg11	2.2 ± 0.2	–	–	–	–	–	–
		Bg11-P	1.8 ± 0.1	–	–	–	–	–	–
	*Chroococcidiopsis* 049	Bg11	1.2 ± 0.0	–	–	0.5 ± 0.0	–	–	–
		Bg11-P	^a^	–	–	–	–	–	–
	*Chroococcus* 055	Bg11	4.0 ± 0.1	–	^a^	1.1 ± 0.0	–	–	–
		Bg11-P	–	–	–	–	–	–	–
	*Fischerella* 067	Bg11	14.5 ± 1.0	–	–	2.0 ± 0.1	–	–	–
		Bg11-P	2.3 ± 0.2	–	–	1.1 ± 0.0	–	–	–
	*Nostoc* 129	Bg11	9.0 ± 0.7	–	–	1.1 ± 0.2	–	–	–
		Bg11-P	–	–	–	–	–	–	–

The impact of the lack of inorganic phosphate on the accessibility of AAPs for microbes was determined (via the Bg11-P medium application). Concentrations are expressed in micromoles per liter (μM) + S.D. (*n* ≥ 3). Initially, all media were fortified with the addition of aminopolyphosphonates at a final concentration of 100 μM. The presented values were determined in a post-cultured media after 14 days of cyanobacteria culturing using the elaborate HPLC method

–Is attributed to not detected

^a^The presence of an intermediate was confirmed in the experimental culture but it did not exist in a 14-day post-culture media; “0.0”—S.D. value below 0.05 μM

Aminotri(methylenephosphonic) acid (ATMP) molecules underwent degradation with a release of aminomethylphosphonic acid (AMPA), regardless of the presence of cyanobacteria and a deficiency in inorganic phosphate. The main metabolite of ATMP was detected in a post-culture media at a concentration of approximately 100 μM. Only in the culture of *Chroococcidiopsis* 049 was this intermediate identified at a lower concentration (41.3 μM), similar to that measured in the substrate control (34.0 μM). Thus, in this case, the formation of AMPA should be considered catalyzed by metal ions and not as an action of cyanobacteria. However, when examined, polyphosphonate was the sole available source of phosphorus, and aminomethylphosphonic acid was present at a concentration of approximately 127 μM. A similar increase in AMPA release in Bg11-P, in comparison to a Bg11 medium, was also noticed for *Nostoc* 129. In both media, *N*-(methyl)aminomethanephosphonic acid (MAMPA) was detected in nearly identical amounts (~ 3 μM). It is consistent with the data presented in Table 5 and indicates the lack of a microbial contribution in ATMP decomposition with the *N*-(methyl)aminomethanephosphonic acid production.

The appearance of MAMPA as the main metabolite in the biodegradation process of *N,N*-bis(phosphonomethyl) glycine (GBMP) has been proven for the first time. In a Bg11 medium, after 2 weeks, its level was approximately 44 μM. However, the cultivation of cyanobacteria in the presence of 100 μM of GBMP led to the enhancement of the MAMPA concentration from 83 μM for *Chroococcus* 055, even up to 156 μM for *Chroococcidiopsis.* It can be stated that the weaker biodegradation rate of GBMP was noticed when it was the sole source of phosphorus. However, we should remember that starved microorganisms induce a number of genes that activate enzymatic pathways that are involved in P acquisition and assimilation (Quinn et al. 2007). Therefore, the detected lower concentration of MAMPA unambiguously does not mean a weaker decomposition of GBMP. The slightly higher AMPA and *N-*phosphomethyl glycine (NPMG) concentrations were measured after microbial treatments compared with a non-inoculated Bg11 medium. However, this is a consequence of chemical breakdown.

It is worth emphasizing that in the case of aminotri(methylenephosphonic) acid (ATMP) and *N,N*-bis(phosphonomethyl) glycine (GBMP) subjected to both microbial and chemical degradation in a Bg11 media, cyanobacteria enhanced the formation of characteristic products of degradation several times, including AMPA for ATMP and MAMPA in the case of GBMP. Therefore, it can be assumed that the final effectiveness of AAP decomposition is related to the parallel action of metal ions and microbial activity. The biodegradation and chemical oxidation products are indicative of C–N and C–P bond cleavage.

Scale inhibitor, diethylenetriamine penta(methylene phosphonic acid) (DTPMP), during the 2 weeks of incubation in a non-inoculated Bg11 medium, released approximately 8 μM of aminomethylphosphonic acid (AMPA). In the cultures of examined microorganisms, the amount of AMPA was lower, with an exception of *Fischerella* 067 in a full growth medium. Additionally, for the four out of five examined strains, small amounts of *N*-(methyl)aminomethanephosphonic (MAMPA) were detected. It is worth emphasizing that the DTPMP molecule has been already proven to be a substrate for cyanobacterial enzymes that originate from freshwater *Anabaena variabilis* cells (Drzyzga et al. 2017), which conceivably indicates that the entire degradation occurred intracellularly.

Interestingly, hexamethylenediamine-*N,N,N′,N′ -* tetrakis(methyl phosphonic acid) (HDTMP) seems unlikely to be stable in cyanobacterial cultures. It has been revealed that the presence of the additional peak of retention time is very close to that of NPMG. Due to the lack of unambiguous identification of its structure, the appearance of this substance is reflected based on its amounts, which are presented in area units (mAU) (Table 7). Bearing in mind the retention time of this substance in relation to other examined compounds, it should be expected that its molecule contains a secondary amine group and a phosphonic acid motif. Therefore, the formation of (3-hydroxypropyl)aminomethylphosphonic acid, as the main product of (bio)degradation of HDTMP, may be postulated. The lower concentration of this compound, which is observed in a Bg11-P medium, should be considered the effect of Pi starvation of cells, rather than the result of a weaker decomposition rate. We postulate the appearance of a similar phenomenon for the GBMP molecule.

**Table 7:** Presence of an unidentified product (rt = 13 min) expressed as an area unit (mAU)

Substrate	Cyanobacteria strain	Quantity of the searched product (mAU)		
		Bg11	*Post*-Bg11	*Post*-Bg11-P
HDTMP	*Anabaena* 007	1.801 ± 0.075	1.429 ± 0.411	0.513 ± 0.043
	*Chroococcidiopsis* 049		2.300 ± 0.115	0.928 ± 0.027
	*Chroococcus* 055		1.885 ± 0.134	0.598 ± 0.119
	*Fischerella* 067		1.708 ± 0.074	0.497 ± 0.057
	*Nostoc* 129		2.145 ± 0.101	0.332 ± 0.144

The amounts of unidentified product were determined after 2 weeks in the media of initial addition of 0.1 mM HDTMP. Quantities were expressed in area units (mAU) + S.D. (*n* ≥ 3)

*Bg11* there was a non-inoculated medium, *post*-*Bg11* and *post*-*Bg11-P* the HPLC analysis was performed for the post-cultured media

The stability of aminopolyphosphonates in water can be monitored by tracking the occurrence of their degradation products. The successful use of adopted and modified chromatographic procedure, which is fast, relatively inexpensive, reproducible, and requires neither expensive detector (MS, MS/MS, TOF/MS, ICP/MS, etc.), systems (e.g., UHPLC) nor time-consuming sample preparation process, has undoubtedly proven that it is possible.

## Conclusions

Growing concerns about the quality of aquatic ecosystems should be associated with the implementation of methods that allow estimation of the environmental fate of aminophosphonate xenobiotics. However, due to lack of appropriate analytical procedures and because of degradation of these compounds, it is not easy to evaluate both the presence of these compounds and their influence on inhabiting biota. Degradation of aminopolyphosphonic acids may be the result of two processes: the catalytic impact of metals ions (Nowack 2002, 2003) and metabolic activity of microorganisms (Drzyzga et al. 2017). Both factors can enhance the final yield of decomposition of phosphonate. An optimized analytical procedure, which is based on derivatization (tosylation) of samples, followed by HPLC separation, has proven to be the proper tool to track this phenomenon. Furthermore, this procedure enables qualitative and quantitative determination of the set of products of degradation of aminopolyphosphonates. It is somehow surprising that, for the very first time, *N*-(methyl)aminomethanephosphonic acid (MAMPA) is postulated to be the product of degradation of APPs since its chemical structure is a part of the substrate molecules. Moreover, it is possible that the compound, which was found as a new product of HDTMP (bio)degradation, can be (3-hydroxypropyl)aminomethylphosphonic acid. Additionally, our results demonstrated that the metabolic activity of freshwater cyanobacteria significantly enhanced the process of APPs degradation. As the result, the concentration of determined products has increased several times in relation to substrate controls, which was reflected by the proportional decrease in the amount of aminopolyphosphonates. However, the environmental fate of aminopolyphosphonic acids, especially the pathways of their (bio)degradation, still requires deeper investigations. The proposed relatively fast and inexpensive analytical procedure creates an opportunity for determining the set of amino derivatives in one step and at a nanomolar level. Hence, its application may enrich the knowledge about alterations in the structures of polyphosphonates that are exposed to the action of microbial communities.
